# Obesity, Physical Function, and Training Success in Community-Dwelling Nonsarcopenic Old Adults

**DOI:** 10.1155/2019/5340328

**Published:** 2019-02-18

**Authors:** O. G. Geirsdottir, M. Chang, P. V. Jonsson, I. Thorsdottir, A. Ramel

**Affiliations:** ^1^The Icelandic Gerontological Research Institute, Reykjavik, Iceland; ^2^Faculty of Food Science and Nutrition, University of Iceland, Reykjavik, Iceland; ^3^School of Education, University of Iceland, Reykjavik, Iceland; ^4^Department of Geriatrics, National University Hospital of Iceland, Reykjavik, Iceland; ^5^School of Health Sciences, University of Iceland, Reykjavik, Iceland; ^6^Unit for Nutrition Research, University of Iceland, Reykjavik, Iceland

## Abstract

**Objectives:**

Obesity-related physiological changes can limit improvements of obese subjects after training. The aim was to investigate obesity, muscular strength, and physical function in community-dwelling nonsarcopenic old adults.

**Methods:**

Nonsarcopenic subjects (*N*=229, 73.7 ± 5.7 years; 21% normal weight, 42% overweight, and 37% obese based on body mass index (BMI)) participated in a 12-week resistance exercise program. Leisure time physical activity (LTPA), body composition (dual-energy X-ray absorptiometry), quadriceps strength (maximum voluntary isometric contraction; absolute and relative to body weight), and physical function in terms of 6-minutes-walk-for-distance (6MWD) and timed up and go (TUG) were measured baseline and endpoint.

**Results:**

At baseline, normal weight participants had lower absolute quadriceps strength (−43 ± 22 N, *P*=0.015) than obese, but better quadriceps strength relative to body weight (1.4 ± 0.7 N/kg, *P* < 0.001), 6MWD (53 ± 27 m, *P* < 0.001), and TUG (−1.4 ± 0.7 sec, *P* ≤ 0.001). LTPA was positively associated with 6MWD and TUG (both *P* < 0.05), but based on general linear models, differences in LTPA between BMI categories did not explain differences in 6MWD and TUG between BMI categories. During the program, dropout (11.9%) and attendance (85%) were similar between BMI groups. After the intervention, body composition and physical function significantly improved in all three BMI categories; however, normal weight participants lost more body fat (−1.53 ± 0.78%, *P*=0.014), gained more lean mass (0.70 ± 0.36 kg, *P* < 0.001) and relative quadriceps strength (0.31 ± 0.16 N/kg, *P*=0.017), and improved more on the 6MWD (24 ± 12 m, *P* < 0.001) but gained less grip strength (−2.4 ± 1.3 N/kg, *P*=0.020) compared to obese. There were no differences in TUG or absolute quadriceps strength changes between the BMI strata. Physical function at baseline as well as training success of overweight participants was located between the normal weight and obesity groups.

**Conclusion:**

Nonsarcopenic obese community-dwelling old adults have lower physical function than their normal weight counterparts. This difference is not explained by lower LTPA. A 12-week resistance exercise program improves body composition and physical function in normal weight, overweight, and obese old adults; however, obese participants experience less favorable changes in body composition and physical function than normal weight individuals. This trial is registered with NCT01074879.

## 1. Introduction

Obesity has been increasing for several decades in Western countries, and an increased prevalence has also been observed in old adults [1]. Obesity is characterized by a changed physiologic milieu, e.g., alterations in insulin sensitivity, sex hormones, and inflammatory cytokines [2, 3], and consequently, higher BMI has been regarded as risk factor for diseases such as cardiovascular diseases, diabetes, musculoskeletal disorders, and some cancers [4–6].

Unfavorable changes in body composition can be seen during ageing, i.e., body fat increases bit by bit while muscle mass decreases [7, 8]. In particular, visceral and intramuscular fat usually increase, thus resulting into low muscle quality, while subcutaneous fat often actually declines with age. As maintenance of muscular strength and physical function is a cornerstone of successful ageing, exercise has been widely recommended to fight the decrease in muscle mass in old adults and it possibly also prevents obesity [9]. Several studies have indicated that various training programs, either alone or in combination with dietary interventions, are effective to improve body composition and physical function in old people [10–12].

However, there are obesity-related physiological changes [13–16], which can potentially limit improvements of obese subjects after training when compared to normal weight subjects. In obese subjects, endocrinological disturbances after strength exercise have been reported, e.g., a blunted growth hormone response or a greater cortisol release. It has been suspected that these hormonal disturbances can lead to a reduced lipolytic response and a decreased skeletal muscle protein synthesis [13]. Accordingly, experimental studies confirmed reduced stimulation of myofibrillar protein synthesis after feeding and resistance exercise in people with obesity when compared with their lean participants [14–16]. Whether reduced muscle protein synthesis measured in short-term experiments actually translates into reduced success after a resistance exercise program of several weeks is currently unknown.

In order to gain knowledge on obesity and its associations with muscular strength and physical function in community-dwelling nonsarcopenic old adults, we conducted this secondary data analysis based on results from a previously published randomized, controlled trial, originally designed to examine the effect of postexercise protein ingestion on the efficacy of strength training [12].

The aim of the present study was to investigate whether (1) nonsarcopenic obese old adults have poorer muscular strength and physical function than their normal and overweight counterparts; (2) this relationship is confounded by leisure time physical activity; and (3) obese old adults show less training success when participating in a 12-week resistance exercise program.

## 2. Methods

### 2.1. Subjects

Subjects (*N*=236) at least 65 years old (range 65–92 years) were invited for participation by advertisements distributed in the capital area of Iceland. The following exclusion criteria were used: low cognitive function (Mini-Mental State Examination (MMSE) ≤19 points) [17], major orthopedic disease, treatment with exogenous testosterone or other medication affecting lean mass, musculoskeletal disorders, or other disorders that could affect their muscle mass. The Icelandic National Bioethics Committee approved the study protocol (VSNb2008060007/03-15). All subjects gave their written, informed consent for study participation. For the present analysis, participants with sarcopenia as defined by the revised European consensus on definition and diagnosis [18] were excluded. All measurements were conducted at baseline (within one week before the intervention started) and endpoint of the study (within one week after the intervention ended).

### 2.2. Intervention

The resistance exercise program has been described in previous publications [12, 19]. In short, it was designed to increase strength and mass of major muscle groups, and the participants exercised 3 days/week for twelve weeks in groups supervised by study staff. A training session included warmup (10–15 minutes), weight lifting (10 different exercises, 3 sets, each exercise was repeated 6–8 times at 75–80% of the 1-repetition maximum; 45–60 minutes), and cool down including stretching (10–15 minutes).

### 2.3. Assessments

#### 2.3.1. Muscular Strength

*(1) Quadriceps Strength*. Quadriceps strength (maximum voluntary isometric contraction (MVIC) in N) was tested using an isokinetic dynamometer (Kin-Com®500H, Chattanooga).

*(2) Grip Strength*. Hand grip strength (lb) was measured using a hydraulic hand dynamometer (Baseline®Baseline Evaluations Corporation).

#### 2.3.2. Physical Function

*(1) Six-Minutes-Walk-for-Distance (6MWD)*. The 6MWD (in minutes) was conducted according to the guidelines from the American Thoracic Society [20].

*(2) Timed Up and Go Test (TUG)*. This test (shown as seconds) was conducted as outlined in the publication from Podsiadlo and Richardson [21].

For more details, see [12] and [19].

#### 2.3.3. Body Composition

Body weight was measured in light underwear on a calibrated scale (model no. 708; Seca, Hamburg, Germany), and height was measured with a calibrated stadiometer (model no. 206; Seca, Hamburg, Germany). BMI was calculated from height and weight (kg/m^2^). Participants were categorized according to their BMI into normal weight (18.5–24.9 kg/m^2^), overweight (BMI 25.0–29.9 kg/m^2^), and obese (BMI ≥ 30 kg/m^2^) [22]. Detailed body composition (body fat (% and kg), lean mass (kg), and appendicular skeletal muscle (kg)) was assessed by dual-energy X-ray absorptiometer (DXA) with Hologic QDR-2000 plus®, Hologic Inc., Waltham, MA, USA.

#### 2.3.4. Leisure Time Physical Activity (LTPA)

Information on LTPA (shown as min/week) during the last 12 months was collected using a questionnaire [23] based on the Compendium of Physical Activities [24] and Paffenberger's questionnaire [25].

#### 2.3.5. Demographic Variables

Background variables (age (years), current smoking (yes vs. no), and alcohol use (yes vs. no)) were assessed using questionnaires.

### 2.4. Statistical Analysis

Statistical analysis was conducted using SPSS for Windows version 24.0 (SPSS, Chicago, IL, USA), and the level of significance was *P* < 0.05. Data were checked for normal distribution using the Kolmogorov–Smirnov test and are shown as mean ± standard deviation (SD).

#### 2.4.1. Baseline Data

Crude comparisons between the three groups were done using 1-way ANOVA including LSD post hoc test. In order to eliminate the potential confounding effects of, e.g., age, uneven gender distribution and different levels of LTPA between the BMI categories, we used linear models (*general linear model–univariate* in SPSS) correcting for these variables to further investigate baseline differences in physical function and muscular strength between the groups: model 1 (intercept, BMI categories, age, and gender) and model 2 (additionally LTPA).

The *general linear model–univariate* procedure in SPSS provides regression analysis and analysis of variance for one dependent variable by one or more factors (which divide the population into groups) and/or variables (continuous covariates). Both balanced and unbalanced models can be tested, and this method is robust to departures from normal distribution [26].

Linear models (*general linear model–univariate)* were also used to find out how much variation either absolute or relative quadriceps strength explains of TUG and 6MWD (dependent variables).

One-way ANOVA including LSD post hoc test was used to investigate whether there was a difference between participants when categorized into six groups according to their BMI status (normal weight, overweight, and obese) and LTPA (active and inactive).

#### 2.4.2. Intervention Effects

In order to investigate changes after training in the three BMI categories, we used linear models (*general linear model–univariate*) correcting for potential confounders (anthropometric variables: corrected for age and gender; muscular strength and physical function changes: corrected for age, gender, and the corresponding baseline value).

## 3. Results

Only two of 236 participants were sarcopenic, and they were excluded from the present analysis. All together, complete data from 229 participants were available. The participants appeared to be healthy, but several had hypertension, hyperlipidemia, or type 2 diabetes [19, 27].

### 3.1. Baseline Function

Baseline characteristics of the participants stratified by BMI are shown in Table 1. Of the participants, 41.5% were male; however, gender distribution was different between BMI strata with a higher proportion of males in the obesity category. Mean time of LTPA was 342 ± 341 minutes/week, and the two most frequent activities reported were walking and gardening. Both normal weight and overweight participants exercised more than obese participants. There were obvious differences in body composition between strata. Normal weight participants had lower quadriceps and grip strength when compared to obese, but better quadriceps strength relative to body weight, TUG, and 6MWD.

When further comparing physical strength and function between the three different BMI strata using linear models controlling for various confounders (Table 2), we found that when correcting for age and gender, differences in grip strength was no longer significant between the groups. However, the differences in the other strength and function variables previously observed in the crude analyses remained significant. LTPA was positively associated with most of the functional variables, but inclusion of LTPA in the models did only marginally change the differences in function between BMI strata.

**Figure 1:**
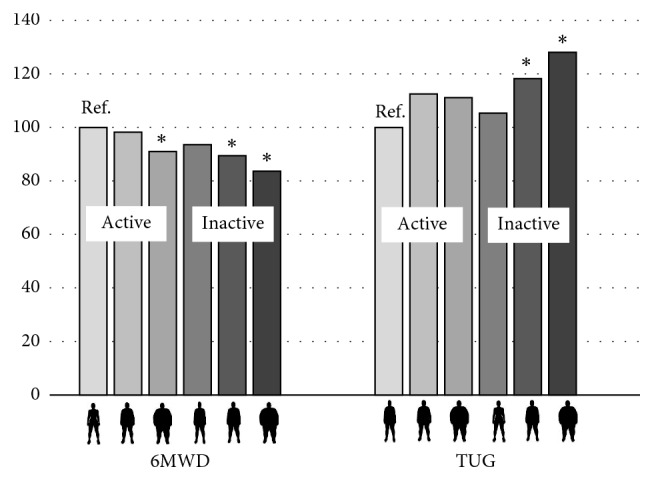
Physical function (6MWD and TUG) according to six categories of BMI (normal weight, overweight, and obese) and LTPA (active and inactive). Values are expressed as % of the reference which is the normal weight-active category. Higher 6MWD and lower TUG indicate better physical function. ^*∗*^Significantly different from the normal weight-active category according to 1-way ANOVA including LSD post hoc test. 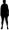

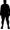

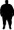
 indicate normal weight, overweight, and obese, respectively.

Figure 1 shows actual physical function of participants stratified by BMI and LTPA (6 groups). The figure indicates that BMI and LTPA are both independently associated with physical function.

Quadriceps strength relative to body weight was stronger related to physical function than absolute quadriceps strength. We found that relative quadriceps strength explained 23% and 29% of the variance observed in TUG and 6MWD, respectively. Accordingly, absolute quadriceps strength explained only 13% and 17% of the variance, respectively.

### 3.2. Training Effects

**Figure 2:**
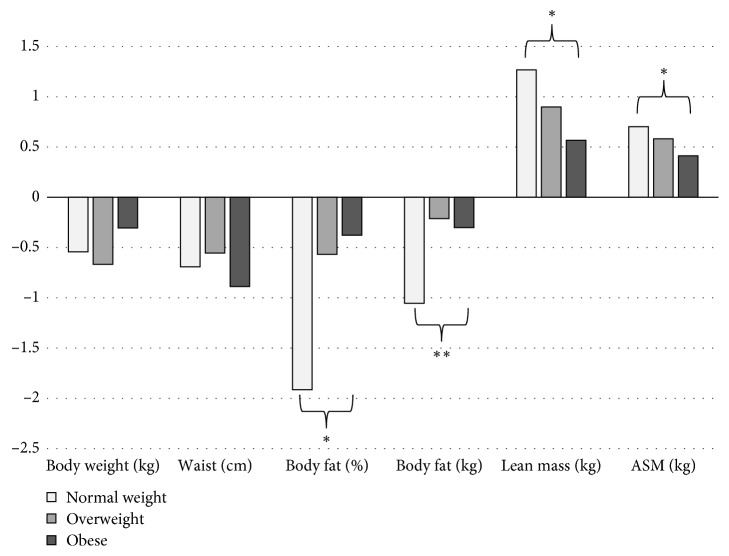
Estimated anthropometric changes after the resistance exercise program in normal weight, overweight, and obese participants. Estimates based on linear models (*general linear model–univariate* in SPSS) corrected for age and gender. ^*∗*^Significant differences between normal weight and obese participants. ^*∗∗*^Borderline significant differences (*P*=0.08) between normal weight and obese participants. ASM, appendicular skeletal muscle.

During the 12-week resistance exercise program, dropout rate and attendance were similar between the three BMI groups (Table 1). The program improved body composition (Figure 2) and physical function of the participants (quadriceps strength: 53.5 ± 52.6 N; relative quadriceps strength: 0.65 ± 0.64 N/kg body weight; grip strength: 3.1 ± 5.6 lb; 6MWD: 33 ± 35 m; TUG: −0.64 ± 1.12 sec; all *P* < 0.001); however, there were significant differences between groups.

**Figure 3:**
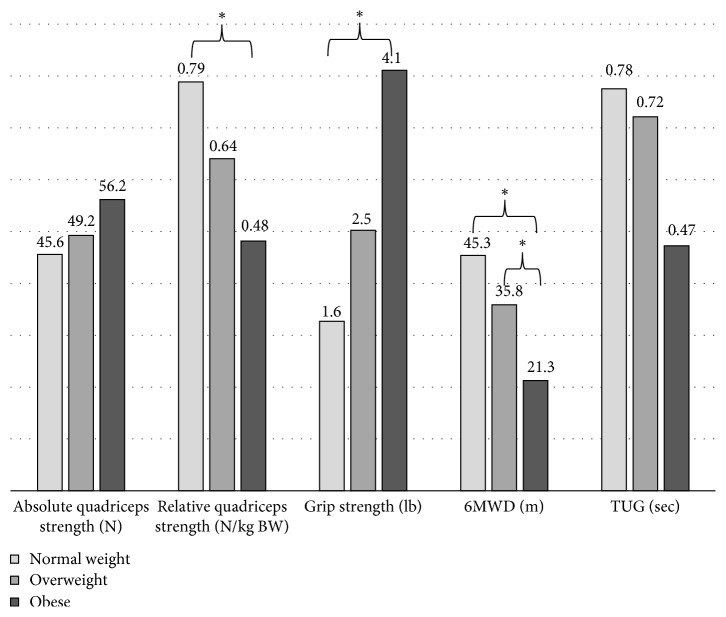
Estimated improvements in muscular strength and physical function after the resistance exercise program in normal weight, overweight, and obese participants. Estimates based on linear models (corrected for age, gender, and the corresponding baseline value) from Table 3. ^*∗*^Significant differences between categories.

Figure 2 shows the estimated means of anthropometric changes for each of the three categories (corrected for age and gender).  Table 3 shows the differences in changes in muscular strength and physical function changes between the three groups (corrected for age, gender, and the corresponding baseline value).  Figure 3 shows the estimated improvements in muscular strength and physical function after the resistance exercise program in normal weight, overweight, and obese participants based on calculations from Table 3.

Normal weight participants lost more body fat and gained more muscle mass and relative quadriceps strength when compared to obese. Further, normal weight participants improved more on the 6MWD but gained less grip strength. There were no differences in improvements in TUG or quadriceps strength between the BMI strata (Table 3).

## 4. Discussion

The present study investigated the associations between obesity, muscular strength, and physical function in nonsarcopenic community-dwelling old adults in Iceland using a cross-sectional as well as a longitudinal approach. At baseline of our study, obese participants had higher absolute quadriceps strength but lower physical function when compared to normal weight participants. After the resistance exercise training program, both normal weight and obese participants improved; however, more favorable changes in body composition and 6MWD were seen in the normal weight group. Both baseline function and training success of overweight participants were located between the normal weight and obesity groups.

### 4.1. Baseline Characteristics and Function

Our participants were subjects who volunteered to take part in a 12-week resistance exercise program. They were physically active, and their average exercise time per day was close to the recommended amount of at least 30 minutes [28]. Apparently, our participants were not representative for this age group, although physical function of our subjects was in accordance with age; i.e., TUG time tended to be at the faster end of the reference spectrum [29] and 6MWD at the corresponding slower end [30].

At baseline, obese participants showed more absolute strength, but part of this was explained by the uneven gender distribution between BMI categories. Relative to body weight, though, normal weight participants were stronger. In our calculations, relative strength was a better predictor of physical function than absolute strength, which is also reflected in the better TUG time and further 6MWD in the normal weight group. LTPA was higher in normal weight participants than in their obese peers, and it was positively related to physical function and strength, but interestingly, the difference in LTPA between the BMI strata did not explain their functional differences.

### 4.2. Resistance Exercise and Training Success

Our resistance exercise program was successful with a low dropout rate, high attendance, and anthropometric as well as functional improvements observed in all three BMI groups. These changes were in agreement with previous published studies [31–33].

In our study, dropout of 11.9% was low compared to earlier reported studies [34–36] and not significantly different BMI categories. There are several potential reasons why dropout was low but we think in particular that our study population consisted of rather healthy volunteers who did neither represent the general population at this age nor a clinical sample of patients. It can be assumed that volunteers show higher motivation and compliance towards physical training independently from the BMI category.

After the 12 weeks, body composition improved more in normal weight than in obese participants as did relative quadriceps strength and gait speed. We did not observe any significant differences for TUG; however, obese individuals gained more grip strength during the intervention. Considering that attendance was similar, we can only speculate on the reasons for these observed differences, but they may be related to insulin resistance, intramuscular fat infiltration, and/or inflammation which are disturbed metabolic features frequently related to obesity [37, 38].

Insulin can stimulate skeletal muscle growth, and studies have shown that the hormone reduces muscle protein breakdown [39], as well as increases muscle protein synthesis [40], and thus, poor insulin sensitivity can have a negative effect on muscle protein homeostasis. Further, it is thought that intramuscular adipose tissue is not only a consequence of increased body fatness or of loss of muscle quality during ageing or physical inactivity [37, 41, 42] but it may also play an active role in affecting muscular function by releasing inflammatory cytokines which results into lower protein synthesis [43] and lower muscle quality [44], factors that all contribute to poorer muscle function and immobility in older adults [45].

Considering that it takes much time and effort to improve body composition and physical function by exercise, maybe combined nutrition-exercise interventions are needed for obese individuals in order to achieve best possible results.

### 4.3. Strengths and Limitations

We think that it is of great importance to investigate nonsarcopenic and obese old adults, as both characteristics are very prevalent in community-dwelling old adults [1, 46], and to our best knowledge, there are currently no studies available, which have compared training success of different BMI categories in old adults. Further, it is a strength of this study that we accessed the participants both cross-sectionally and longitudinally, thus getting a more complete idea on physical function status and training success in old obese adults. Since many previous studies on obesity and physical function have focused on sarcopenic obesity, we think it is of importance that we only included nonsarcopenic individuals in this data analysis because the majority of old adults in the community is not sarcopenic. To our best knowledge, there are no previous studies that have compared training success in normal weight, overweight, and obese old adults.

It is a limitation of our study that our participants were highly active volunteers in an intervention study, and thus, they do not present the typical population of this age group which makes it inappropriate to generalize the findings.

## 5. Conclusions

Nonsarcopenic community-dwelling old adults who are obese have lower physical function than their normal weight counterparts, and this difference is not explained by lower LTPA. A 12-week resistance exercise program results into improvements in body composition and physical function in normal weight, overweight, and obese old adults; however, obese participants experience less favorable changes in body composition and physical function than normal weight individuals.

## Figures and Tables

**Table 1:** Characteristics of the participants.

Descriptives	All subjects	Normal weight^*∗*^	Overweight^*∗*^	Obese^*∗*^	
	(*N*=229)	(*N*=48)	(*N*=96)	(*N*=85)	
	Mean ± SD	Mean ± SD	Mean ± SD	Mean ± SD	*P* value^*∗*^
Age (years)	73.5 ± 5.7	74.1 ± 5.8	73.2 ± 6.2	73.3 ± 4.8	n.s.
Male (%)	41.5	27.5	38.5	52.9	0.011
Smokers (yes in %)	6.0	5.9	4.2	8.3	n.s.
Alcohol (yes in %)	81.5	80.4	85.1	78.8	n.s.
LTPA (min/week)	342 ± 342	445 ± 392	377 ± 351	252 ± 277	0.003^1,3^
Dropout (%)	11.9	5.9	13.5	9.4	n.s.
Attendance (%)	85.8 ± 20.2	87.1 ± 17.7	83.0 ± 23.5	88.1 ± 17.1	n.s.
Body weight (kg)	82.6 ± 17.5	64.9 ± 7.1	78.2 ± 10.9	98.1 ± 14.9	<0.001^1,2,3^
Waist circumference (cm)	99.8 ± 14.5	84.4 ± 8.4	96.7 ± 8.7	112.9 ± 11.0	<0.001^1,2,3^
BMI (kg/m^2^)	28.8 ± 4.8	23.0 ± 1.5	27.3 ± 1.5	33.9±	<0.001^1,2,3^
Body fat (%)	38.2 ± 7.3	33.5 ± 8.3	37.4 ± 6.0	42.1 ± 5.8	<0.001^1,2,3^
Body fat (kg)	31.7 ± 9.9	21.7 ± 5.2	29.0 ± 4.7	41.2 ± 8.4	<0.001^1,2,3^
Lean mass (kg)	47.9 ± 10	42.0 ± 7.0	46.3 ± 9.2	53.3 ± 9.8	<0.001^1,2,3^
Appendicular skeletal muscle (kg)	24.4 ± 5.4	20.8 ± 3.2	23.7 ± 5.1	27.5 ± 5.1	<0.001^1,2,3^
Quadriceps strength (N)	465 ± 124	417 ± 97	464 ± 119	496 ± 135	0.002^1,2^
Relative quadriceps strength (N/kg BW)	5.7 ± 1.4	6.4 ± 1.2	5.9 ± 1.3	5.1 ± 1.4	<0.001^1,2,3^
Grip strength (lb)	62.5 ± 19.1	56.3 ± 15.4	63.0 ± 20.1	65.9 ± 19.4	0.020^1,2^
6MWD (m)	454 ± 79	479 ± 73	465 ± 81	426 ± 74	<0.001^1,3^
TUG (sec)	7.9 ± 2.2	7.1 ± 1.3	8.0 ± 2.6	8.4 ± 2.0	0.003^1,2^

^*∗*^One-way ANOVA including LSD post hoc test; ^1^normal weight vs. overweight significant difference; ^2^normal weight vs. obese significant difference; ^3^obese vs. overweight significant difference.

**Table 2:** Comparison in baseline function between normal weight, overweight, and obese participants using linear models^*∗*^.

Parameter estimates dependent variable	Parameter	Model 1				Model 2			
		*B*	95% CI		*P* value	*B*	95% CI		*P* value
Quadriceps strength (N)	Intercept	1050.87	885.163	1216.58	<0.001	1007.37	838.628	1176.11	<0.001
	Normal weight	−34.949	−69.025	−0.874	**0.044**	−43.145	−77.696	−8.594	**0.015**
	Overweight	−10.293	−39.264	18.679	0.484	−14.575	−43.537	14.387	0.322
	Obese	Ref.				Ref.			
	Age (years)	−8.629	−10.901	−6.358	<0.001	−8.186	−10.472	−5.901	<0.001
	Male^1^	143.809	117.445	170.173	<0.001	143.484	117.358	169.610	<0.001
	LTPA^2^ (min/week)					0.042	0.004	0.080	0.028
		*R* ^2^ = 43.4%				*R* ^2^ = 46.1%			

Rel. quadriceps str. (N/kg BW)	Intercept	9.713	7.518	11.908	<0.001	9.125	6.891	11.359	<0.001
	Normal weight	1.511	1.060	1.963	**<0.001**	1.401	0.943	1.858	**<0.001**
	Overweight	0.921	0.537	1.305	**<0.001**	0.863	0.480	1.247	**<0.001**
	Obese	Ref.				Ref.			
	Age (years)	−0.068	−0.098	−0.038	<0.001	−0.062	−0.092	−0.032	<0.001
	Male	0.716	0.367	1.065	<0.001	0.711	0.366	1.057	<0.001
	LTPA (min/week)					0.001	<0.001	0.001	0.025
		*R* ^2^ = 22.8%				*R* ^2^ = 26.1%			

Grip strength (lb)	Intercept	108.052	88.780	127.324	<0.001	105.186	85.406	124.966	<0.001
	Normal weight	−0.888	−4.851	3.075	0.659	−1.428	−5.478	2.622	0.488
	Overweight	0.691	−2.678	4.061	0.686	0.409	−2.986	3.804	0.812
	Obese	Ref.				Ref.			
	Age (years)	−0.809	−1.073	−0.545	<0.001	−0.780	−1.048	−0.512	<0.001
	Male	32.322	29.255	35.388	<0.001	32.300	29.238	35.363	<0.001
	LTPA (min/week)					0.003	−0.002	0.007	0.216
		*R* ^2^ = 68.1%				*R* ^2^ = 69.1%			

6MWD (m)	Intercept	968.333	860.340	1076.33	<0.001	912.846	806.580	1019.11	<0.001
	Normal weight	62.965	40.758	85.173	**<0.001**	52.512	30.753	74.271	**<0.001**
	Overweight	45.106	26.226	63.987	**<0.001**	39.644	21.405	57.883	**<0.001**
	Obese	Ref.				Ref.			
	Age (years)	−7.573	−9.053	−6.093	<0.001	−7.008	−8.447	−5.569	<0.001
	Male	30.925	13.744	48.107	<0.001	30.511	14.058	46.964	<0.001
	LTPA (min/week)					0.054	0.030	0.077	<0.001
		*R* ^2^ = 36.5%				*R* ^2^ = 44.0%			

TUG (sec)	Intercept	−6.690	−9.501	−3.879	<0.001	−5.655	−8.485	−2.825	<0.001
	Normal weight	−1.533	−2.111	−0.955	**<0.001**	−1.338	−1.917	−0.758	**<0.001**
	Overweight	−0.529	−1.021	−0.038	**0.035**	−0.427	−0.913	0.058	0.084
	Obese	Ref.				Ref.			
	Age (years)	0.208	0.170	0.247	<0.001	0.198	0.159	0.236	<0.001
	Male	−0.483	−0.930	−0.036	0.034	−0.475	−0.913	−0.037	0.034
	LTPA (min/week)					−0.001	−0.002	<0.001	0.002
		*R* ^2^ = 32.2%				*R* ^2^ = 41.5%			

^*∗*^Linear models (*general linear model–univariate* in SPSS) to investigate baseline differences in physical function and muscular strength between the groups. Model 1: intercept, BMI categories, age, and gender; model 2: additionally LTPA. ^1^as compared to female; ^2^LTPA, leisure time physical activity.

**Table 3:** Changes^*∗*^ in strength and physical function after a resistance exercise training program in normal weight, overweight, and obese participants.

Parameter estimates dependent variable	Parameter	*B*	95% CI		*P* value
Quadriceps strength (N)	Intercept	273.363	148.539	398.187	<0.001
	Male	19.712	1.181	38.243	0.037
	Normal weight	−10.576	−29.920	8.769	0.282
	Overweight	−6.924	−23.160	9.312	0.401
	Obese	Ref.			
	Age (years)	−2.384	−3.856	−0.912	0.002
	Baseline quad. strength	−0.104	−0.181	−0.027	0.008

Rel. quadriceps str. (N/kg BW)	Intercept	2.735	1.405	4.066	<0.001
	Male	0.013	−0.172	0.199	0.888
	Normal weight	0.307	0.054	0.560	**0.017**
	Overweight	0.159	−0.046	0.365	0.127
	Obese	Ref.			
	Age (years)	−0.022	−0.038	−0.005	0.010
	Baseline rel. quad. strength	−0.107	−0.177	−0.038	0.003

Grip strength (lb)	Intercept	28.282	15.938	40.625	<0.001
	Male	3.499	0.756	6.242	0.013
	Normal weight	−2.420	−4.456	−0.384	**0.020**
	Overweight	−1.542	−3.265	0.181	0.079
	Obese	Ref.			
	Age (years)	−0.259	−0.405	−0.114	0.001
	Baseline grip strength (lb)	−0.105	−0.176	−0.034	0.004

6MWD (m)	Intercept	214.698	119.155	310.240	<0.001
	Male	4.029	−5.921	13.979	0.426
	Normal weight	24.124	10.737	37.511	**<0.001**
	Overweight	14.586	3.349	25.823	**0.011**
	Obese	Ref.			
	Age (years)	−1.549	−2.564	−0.534	0.003
	Baseline 6MWD (m)	−0.175	−0.255	−0.096	<0.001

TUG (sec)	Intercept	−1.609	0.419	−3.637	0.119
	Male	0.058	0.369	−0.252	0.711
	Normal weight	−0.303	0.124	−0.729	0.163
	Overweight	−0.249	0.094	−0.592	0.154
	Obese	Ref.			
	Age (years)	0.047	0.081	0.014	0.006
	Baseline TUG (sec)	−0.306	−0.196	−0.415	<0.001

^*∗*^Based on linear models (*general linear model–univariate* in SPSS) correcting for age, gender, and the corresponding baseline value.

## Data Availability

Data are not available due to laws of the Icelandic Data Protection Authority.
